# Physiological and biochemical variations in different pepper (*Capsicum annuum* var. *conoides*) varieties under salt stress

**DOI:** 10.1186/s12870-025-07873-0

**Published:** 2025-12-08

**Authors:** Xinru Li, Yamin Zhang, Doudou Zhang, Junhao Liu, Nan Xu, Yike Zhong, Xu Han, Zhuona Chen, Yingpeng Hua, Nan Lu, Bo Li, Yinggang Wang, Wenyue Li, Haihong Shang

**Affiliations:** 1 Present Address: Shool of Agriculture and Biomanufacturing, Zhengzhou University, Zhengzhou, 450001 China; 2 Henan OULAND Seed Industry Co., Ltd, Zhengzhou, 450003 China; 3 Institute of Cotton Research, Chinese Academy of Agricultural Sciences, Anyang, 455000 Henan China; 4 Ningling County Bureau of Agriculture and Rural Affairs, Shangqiu, 476000 Henan China

**Keywords:** Pod pepper, Salt stress, Physiological profiling, Transcriptomics, Metabolomics

## Abstract

**Background:**

Pepper (*Capsicum annuum* L.), an annual herbaceous plant of the Solanaceae family, is extensively cultivated as both a fresh vegetable and a condiment, ranking among the most widely grown vegetable crops globally. Pod pepper (*Capsicum annuum* var. *conoides*), major variety of pepper, often suffers from salt stress during growth, leading to reduced yield and quality. However, systematic multi-omics studies on the salt stress response in pod pepper remain limited.

**Results:**

To elucidate physiological responses and salt tolerance mechanisms in pepper plants, this study screened 63 pod pepper accessions and selected salt-tolerant (P47) and salt-sensitive (P18) varieties for comparative analysis. Integrated physiological, transcriptomic and metabolomic analyses revealed that P47 exhibited lower leaf relative electrical conductivity, malondialdehyde content, and Na⁺/K⁺ ratio, along with better maintenance of chlorophyll a content and antioxidant enzyme activities. Transmission electron microscopy showed that P47 maintained intact mesophyll cell ultrastructure under stress, while P18 exhibited severe damage, including chloroplast membrane disintegration and starch grain degradation. Integrated transcriptomic and metabolomic analysis further revealed significant enrichment of differential metabolites in pathways such as phenylpropanoid biosynthesis, tyrosine metabolism, alkaloid biosynthesis, and glycerophospholipid metabolism. Key salt-responsive genes, including *SAUR*, *ARF7*, and *TAT*, involved in plant hormone signal transduction, tyrosine metabolism, and alkaloid biosynthesis, were identified.

**Conclusions:**

This study, for the first time, systematically reveals the involvement of the alkaloid pathway in the salt stress response of pod pepper through integrated multi-omics approaches. It elucidates the physiological and molecular mechanisms by which the salt-tolerant variety enhances antioxidant capacity, regulates ion homeostasis, and maintains cellular structural integrity under salt stress. These findings provide a theoretical basis and genetic resources for targeted breeding of salt-tolerant pepper cultivars.

**Supplementary Information:**

The online version contains supplementary material available at 10.1186/s12870-025-07873-0.

## Introduction

Salinity [1], temperature fluctuations [2], drought [3], waterlogging [4], and heavy metals [5], as abiotic stresses, severely constrain crop growth and yield, potentially reducing plant productivity by 50 % to 70 % [6]. Among these, salt stress stands as a major abiotic stress that constrains agricultural development and crop yield [7]. Globally, approximately 20 % of total cultivated land and 33 % of irrigated farmland are affected by salinity, leading to an average yield reduction of over 50 % in major food crops [8]. The ongoing intensification of soil salinization is primarily driven by climate change and anthropogenic activities. Global warming increases evaporation, promoting salt accumulation in soils. Furthermore, improper agricultural practices, such as irrigation and fertilization, significantly exacerbate secondary soil salinization [9]. Projections indicate that by 2050, nearly half of the world's arable land could be affected by salinity [10]. Elevated soil salinity imposes multiple stresses on plants, severely impairing their growth and development, ultimately constraining global crop production and causing significant economic impacts.

Salt stress refers to physiological damage caused primarily by neutral salts such as NaCl and Na₂SO₄, which significantly impairs seedling growth. Upon exposure to high-salinity soil, seedlings initially undergo osmotic stress. This reduces water uptake by roots and impairs nutrient acquisition, leading to ion imbalance and physiological drought. Simultaneously, excessive accumulation of Na⁺ and Cl⁻ ions within seedlings causes ionic toxicity, triggering secondary stresses such as oxidative stress, which disrupts normal physiological and biochemical processes [11]. When salt stress induces ionic and osmotic stress, overproduction of reactive oxygen species (ROS) occurs in subcellular compartments, including the cell wall, peroxisomes, mitochondria, and chloroplasts. The overaccumulation of ROS overwhelms the antioxidant defense system, leading to oxidative stress [12]. Excessive ROS levels cause lipid peroxidation of membranes, disrupt protein structure and function, and inhibit cellular metabolism, ultimately impeding plant growth [13, 14].

Plant salt tolerance is a polygenic trait with abundant quantitative variations. In response to salt stress, plants have evolved complex regulatory mechanisms. During the early stages of salt stress, osmotic adjustment is a key mechanism for maintaining cellular water balance [15]. This mechanism comprises two main components: On one hand, plants selectively absorb and accumulate inorganic ions to enhance cellular turgor pressure and prevent excessive water loss. For instance, in salt-tolerant asparagus (*Asparagus officinalis* L.), reduced Na⁺ uptake and maintenance of a high intracellular K⁺/Na⁺ ratio is crucial for sustaining normal physiological activities [16]. On the other hand, plants synthesize compatible solutes such as free proline to lower intracellular osmotic potential, which helps mitigate ionic toxicity and maintain cellular function. Concurrently, plants employ an intrinsic ROS scavenging system where antioxidant enzymes including superoxide dismutase (SOD) and peroxidase (POD) work synergistically to eliminate surplus ROS and maintain cellular redox homeostasis [17, 18]. This mechanism is further supported by evidence that overexpression of the *AsA* synthase gene in *Arabidopsis thaliana* enhances oxidative stress tolerance through maintaining ROS homeostasis [19]. Pepper (*Capsicum annuum* L.), an annual herbaceous species in the Solanaceae family, is a widely cultivated and important global vegetable crop, consumed both fresh and as a condiment. Variations in climate, geography, and cultivation practices across regions result in considerable morphological diversity, pungency, and flavor profiles among pepper varieties. Pod pepper (*Capsicum annuum* var. *conoides*) is one such distinctive variety. Pod pepper, an often-cross-pollinated plant in the Solanaceae family, is characterized by small fruit size, high pungency, a capsule fruit type, and erect fruit orientation. It is rich in capsaicin, capsanthin, and vitamin C [17], and is consumed fresh, pickled, or dried, receiving considerable market appreciation. With its extended industrial chain and significant value-added potential, the cultivation demand for pod pepper has been steadily increasing. However, pod pepper cultivation is frequently challenged by salt stress, which increases susceptibility to pests and diseases, reduces yield, and results in significant economic losses [18, 19]. Consequently, breeding salt-tolerant pepper varieties and elucidating the mechanisms of pepper salt tolerance are crucial for sustainable pepper production.

Recent advances in transcriptomics and metabolomics have significantly elucidated molecular mechanisms of plant salt tolerance [20]. For instance, comparative transcriptomic analysis of salt-tolerant and salt-sensitive soybean (*Glycine max*) varieties revealed that tolerant genotypes possess a greater number of highly expressed salt-responsive genes [21]. In pepper crops, multi-omics approaches have identified key salt tolerance pathways related to ion homeostasis, ROS scavenging, and hormone signaling transduction in *Capsicum annuum* and *Capsicum chinense* [18, 22]. However, the systematic stress response mechanisms of pod pepper, an economically significant yet salt-sensitive crop, remain unexplored.

This research gap primarily stems from two factors: pod pepper has received limited attention in fundamental research as a regional specialty crop, and its unique physiological responses to salt stress have not been systematically characterized. To address this knowledge gap, we employed an integrated approach combining physiological indicators with transcriptomic and metabolomic analyses to elucidate the salt tolerance regulatory mechanisms in this species. Our methodology involved screening 63 accessions to identify salt-tolerant germplasm, followed by comprehensive analysis of dynamic physiological and biochemical responses under salt stress, ultimately revealing key molecular regulatory networks. These findings provide both a theoretical foundation for breeding stress-resistant pepper varieties and technical strategies for utilizing saline-alkaline lands, thereby supporting sustainable production of high-quality peppers.

## Materials and methods

### Plant growth and treatment

The pepper seeds used in this study were provided by Henan OULAND Seed Industry Co., Ltd. The pepper seeds were soaked in water and subjected to germination at 25 ℃ for 3 days. Subsequently, the uniformly germinated seedlings were transferred to 10 L of half-strength Hoagland’s nutrient solution (pH = 5.8) and cultivated under conditions of 25 ℃, a light intensity of 20,000 lux, with a 16-hour light/8-hour dark photoperiod. The concentration of 150 mmol/L NaCl was selected based on the salt stress methodology described by Erol et al. (2025) and supported by statistical assessment of preliminary experiments, which confirmed its effectiveness in inducing significant varietal differentiation [23]. Salt stress treatment was initiated at the four-leaf stage using a nutrient solution containing this specific concentration.

### Screening of salt-tolerant varieties of pod pepper

Sixty-three pod pepper varieties (Table S1) were collected and subjected to salt stress treatment. The most pronounced phenotypic differences were observed after 6 days of stress treatment. Referring to the evaluation and identification method for salt tolerance in peppers [24], the salt injury index was calculated to preliminarily screen for salt-tolerant and salt-sensitive materials. Plant height (PHt), fresh weight (FW), and dry weight (DW) were measured with ten plants per replicate, and the experiment was conducted in six biological replicates. Based on the salt tolerance coefficient formula, relative plant height (RPHt), relative fresh weight (RFW), and relative dry weight (RDW) were calculated. The membership function method was employed to compute the comprehensive salt-tolerance evaluation value (D value) of salt tolerance for different pod pepper varieties [25], enabling the selection of extremely salt-tolerant and salt-sensitive materials.

### Determination of Salt Tolerance Index (STI)

On the 6th day of salt stress treatment, PHt, FW, and DW of P47 and P18 pod pepper varieties were measured. After obtaining these measurements, the salt tolerance index (STI) for both pod pepper varieties was calculated using the following formula [26].

$$\text{Salt tolerance index}=\mathrm{Salt}-\text{treated value}/\text{Control value }$$

### Determination of physiological indicators of P18 and P47 under salt stress

Fresh pepper leaves collected at 0, 1, 3, and 6 days after salt stress treatment were immersed in ultrapure water and incubated at room temperature for 3–4 hours. The initial conductivity (S1) was recorded, and then the samples were boiled and cooled to room temperature to measure the final conductivity (S2). Following the measurements of S1 and S2, the relative electrolyte leakage was calculated as (S1/S2) × 100 % to quantify membrane integrity [27]. For ion concentration analysis, seedlings of P47 and P18 varieties treated for 6 days were analyzed using an Agilent 7900 inductively coupled plasma mass spectrometry (ICP-MS) system. For transmission electron microscopy (TEM) observation, leaf samples (1 mm^3^) from control and stress-treated P47 and P18 plants at 6 days were subjected to primary fixation with 2.5 % glutaraldehyde, post-fixation with 1 % osmium tetroxide, gradient dehydration with acetone, and epoxy resin infiltration and embedding. Ultrathin sections were then prepared and stained with uranyl acetate and lead citrate.

The proline (Pro) content was determined using the acidic ninhydrin method, which is based on the formation of a red compound upon reaction with ninhydrin under acidic conditions [28]. Superoxide anion (O₂•⁻) levels were measured by the hydroxylamine method, which relies on the reaction of O₂•⁻ with hydroxylamine to form nitrite, followed by colorimetric quantification [29]. Hydrogen peroxide (H₂O₂) concentration was detected via the titanium sulfate method, utilizing the formation of a stable yellow complex between H₂O₂ and titanium (IV) ions in an acidic medium [30]. Malondialdehyde, a marker of lipid peroxidation, was quantified by the thiobarbituric acid (TBA) method, which detects TBA-reactive substances [31]. Additionally, the activities of catalase, superoxide dismutase, and peroxidase were assayed using commercial enzyme activity assay kits (Michy, China) following spectrophotometric methods [32].

Leaf samples were collected from pepper seedlings after 6 days of stress treatment for chlorophyll content determination. Leaves were finely ground, and 0.2 g of tissue was weighed into a 50 mL centrifuge tube. Then, 25 mL of 95 % (v/v) ethanol was added, and the tube was incubated in darkness for 24 h. After centrifugation at 8,000 × g for 10 min, the supernatant was collected, and absorbance at 470, 649, and 665 nm was measured using a UV-Vis spectrophotometer. Chlorophyll content was calculated using the following equations:

$$\text{Chl a}=13.95\times {{A}}_{\mathit{665}}-6.88\times {{A}}_{\mathit{649}}$$

$$\text{Chl b}=24.96\times {{A}}_{\mathit{649}}-7.32\times {{A}}_{\mathit{665}}$$

$$\text{Chl a}+\mathrm{b}=\text{Chl a}+\text{Chl b}$$

Where: Chl a and Chl b represent chlorophyll a and b concentrations (mg/L) in ethanol extract. *A₆₆₅* and *A₆₄₉* are absorbance values at 665 nm and 649 nm, corresponding to the characteristic absorption peaks of chlorophyll a and b. Total Chl is the sum of Chl a and Chl b.

All physiological parameters, measured with three biological replicates of fifteen seedlings each, were analyzed by two-way ANOVA with Sidak's post-hoc test (α = 0.05).

### Transcriptomics determination and analysis

After 6 days of salt stress, transcriptome sequencing was conducted on shoot and root tissues from P47, P18, and their respective untreated controls. The experiment followed a completely randomized design with three biological replicates (*n*=3) per treatment group, with each biological replicate comprising three individual samples. RNA was extracted using the Plant Polysaccharide & Polyphenol-rich RNA Kit (TIANGEN, China), and sequencing libraries were constructed for Illumina Novaseq X plus platform. Base calling was performed using bcl2fastq (v2.20), and raw reads were filtered to obtain clean reads. The clean reads were aligned to the *Capsicum* reference genome (RefSeq Assembly ID: GCF_000710875.1). Gene functions were annotated using the following databases: Nr (NCBI non-redundant protein sequences), Nt (NCBI non-redundant nucleotide sequences), Pfam (Protein family), KOG/COG (Clusters of Orthologous Groups of proteins), Swiss-Prot (a manually annotated and reviewed protein sequence database), KO (KEGG Ortholog database), and GO (Gene Ontology). Transcriptomic analysis was performed on shoot and root tissues of P18 and P47, along with their corresponding control groups. Four comparison groups were established: P18S_VS_CK18S, P18R_VS_CK18R, P47S_VS_CK47S, and P47R_VS_CK47R (S: shoot; R: root). Gene expression levels were quantified using both FPKM (Fragments Per Kilobase of transcript per Million mapped reads) and raw read counts. Differential expression analysis was performed on the raw counts using DESeq2 (v1.16.1), with a significance threshold of an adjusted *P*-value (FDR) < 0.05 and |log2(foldchange)| ≥ 1. FPKM values were utilized for cross-sample comparisons. Finally, GO and KEGG enrichment analyses of the DEGs were performed using the clusterProfiler R package (v3.4.4) [33]. For validation, ten randomly selected differentially expressed genes were analyzed by reverse transcription quantitative real-time PCR (RT-qPCR) (Table 1) using primers designed with Primer 5 (Table S5) and *CaUBI* as the reference gene. The relative expression of target genes was calculated using the 2^(-ΔΔCt) method below. Differences between the treatments were analyzed using Student’s t test. All the experiments were conducted at least three times. The expression stability of the *CaUBI* reference gene was confirmed [34].

**Table 1:** RT-qPCR reaction system

Reagent	Volume
2 × ChamQ Universal SYBR qPCR Master Mix*	10 μL
Primer1	0.8 μL
Primer2	0.8 μL
cDNA	1 μL
RNase-free ddH_2_O	up to 20 μL

$$\Delta \mathrm{Ct}=\mathrm{Ct}\_\mathrm{target}-\mathrm{Ct}\_\mathrm{reference}$$

$$\Delta \Delta \mathrm{Ct}=\Delta \mathrm{Ct}\left(\text{experimental group}\right)-\Delta \mathrm{Ct}\left(\text{control group}\right)$$

$$\text{Relative expression}=2^{\wedge}\left(\Delta\Delta\mathrm{Ct}\right)$$

### Non-targeted metabolomics determination and analysis

After 6 days of stress, the aboveground and root tissues of P47, P18 and untreated control plants were collected, ground to powder, and aliquoted by weight. The experiment included six biological replicates (*n*=6) with six individual samples per group. Pre-chilled methanol extraction solvent was added, followed by thorough vortex mixing. The extracts were maintained at −20 ℃ for 24 h, then centrifuged at 12,000 × g for 15 min, and the resulting supernatant was vacuum-dried. For MS analysis, the samples were reconstituted in 100 μL of acetonitrile-water solution, vortexed, centrifuged, and the supernatant was injected for LC-MS analysis. Metabolite separation was performed on an Agilent 1290 Infinity UHPLC system equipped with equipped with an Agilent ZORBAX Eclipse Plus C18 column (2.1 × 100 mm, 1.8 μm) using a gradient elution program: mobile phase A was water (with 0.1 % formic acid) and B was acetonitrile (with 0.1 % formic acid); the gradient program was: 0–1 min, 2 % B; 1–15 min, increase to 100 % B; 15–18 min, hold at 100 % B; 18–18.1.1 min, decrease to 2 % B; 18.1–20.1 min, re-equilibration. MS/MS spectra were acquired using an AB Triple TOF 6600 mass spectrometer. Differentially accumulated metabolites (DAMs) were identified for the four tissue-specific comparison groups (P18S_VS_CK18S, P18R_VS_CK18R, P47S_VS_CK47S, P47R_VS_CK47R) by integrating univariate analysis (Student's t-test) and Orthogonal partial least squares discriminant analysis (OPLS-DA), with screening thresholds of VIP ≥1, adjusted *p*-value < 0.05, and |log₂FC| ≥ 1 [35]. DAMs were annotated using the HMDB database (https://hmdb.ca/metabolites) and subjected to KEGG pathway enrichment analysis (https://www.genome.jp/kegg/pathway.html). The significance of the enrichment was determined using hypergeometric test *p*-values. Based on significant variations (*p* < 0.05, fold change >2) in abundance of differential metabolites among experimental groups, seven key metabolites were identified. Pearson correlation analyses (|R| >0.8, FDR < 0.05) were conducted between these metabolites and DEGs, followed by the construction of a metabolite-gene co-regulation network (edge weight >0.8) using Gephi software (v.0.10.1).

## Results

### Extremely stringent material selection

Seedlings of diverse pod pepper accessions were subjected to 150 mmol/L NaCl for 6 days. Salt damage indices were calculated from morphological assessments (Table S2) and ranked, identifying four salt-tolerant (P47, P48, P52, P63) and four salt-sensitive materials (P6, P15, P18, P39). A secondary stress treatment confirmed extreme tolerance/sensitivity. Salt-tolerant accessions resumed normal growth after 6 days, with P47 exhibiting optimal recovery. In contrast, salt-sensitive materials displayed severe lateral root necrosis and leaf curling, chlorosis, and withering; P18 showed the most pronounced symptoms (Fig. S1).

**Fig. 1:**
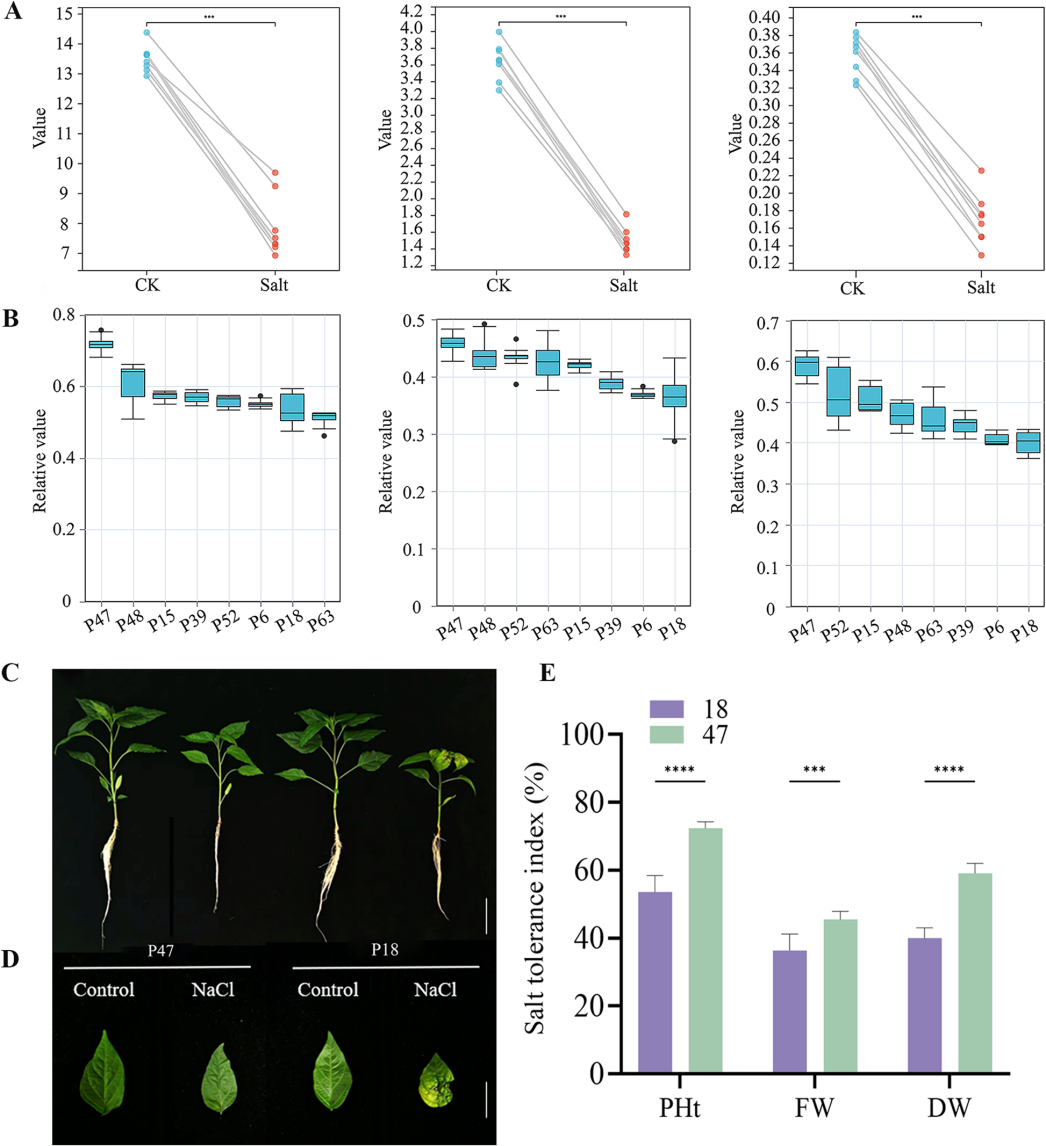
**A** Paired point plots of PHt, FW, and DW for eight pod pepper cultivars in control and treatment groups. **B** Box plots of RPHt, RFW, and RDW for eight pod pepper cultivars. **C** The phenotypes of P47 and P18. Scale bar = 5 cm. **D** The phenotypes of the leaves. Scale bar = 3 cm. **E** Salt tolerance index of PHt, FW, and DW. Data are presented as mean ± SD (*n*=6). Significant differences between varieties were determined by Student's t-test (***P < 0.001, ****P < 0.0001)

Measurements of PHt, FW, and DW revealed significantly higher relative values in P47 and the lowest in P18 (Fig. 1A, B). Materials were ranked by the composite salt tolerance coefficient (D). P47 demonstrated the highest tolerance (D = 1.0), while P18 was the most sensitive (D = 0.0413) (Table 2). Based on integrated phenotypic and quantitative data, P47 (salt-tolerant) and P18 (salt-sensitive) were selected for subsequent experiments.

**Table 2:** Comprehensive salt tolerance analysis of eight pod pepper cultivars

Variety	μ(RPHt)	μ(RFW)	μ(RDW)	D value	Sort
P47	1	1	1	1	1
P48	0.6237	0.8247	0.3539	0.6007	2
P52	0.2281	0.7464	0.6238	0.5327	3
P15	0.2986	0.615	0.566	0.4932	4
P63	0	0.6798	0.3106	0.3301	5
P39	0.2821	0.2737	0.2309	0.2622	6
P6	0.1977	0.0625	0.0454	0.1019	7
P18	0.1240	0	0	0.0413	8

*RPHt* Relative plant height, *RFW* Relative fresh weight, *RDW* Relative dry weight, *D value* Comprehensive salt-tolerance evaluation value

Both P47 and P18 experienced growth inhibition after 6 days of salt stress, with P18 exhibiting more severe effects. P18 leaves showed significant shrinkage, yellowing, and necrosis, whereas P47 leaves displayed only minor shrinkage (Fig. 1C, D). The salt-tolerance indices (STIs) for PHt, FW, and DW were significantly higher in P47 than P18 (Fig. 1E). These results could be evidence that P47 is salt-tolerant, while P18 is salt-sensitive.

### Analysis of physiological indicators of P18 and P47 under salt stress

**Fig. 2:**
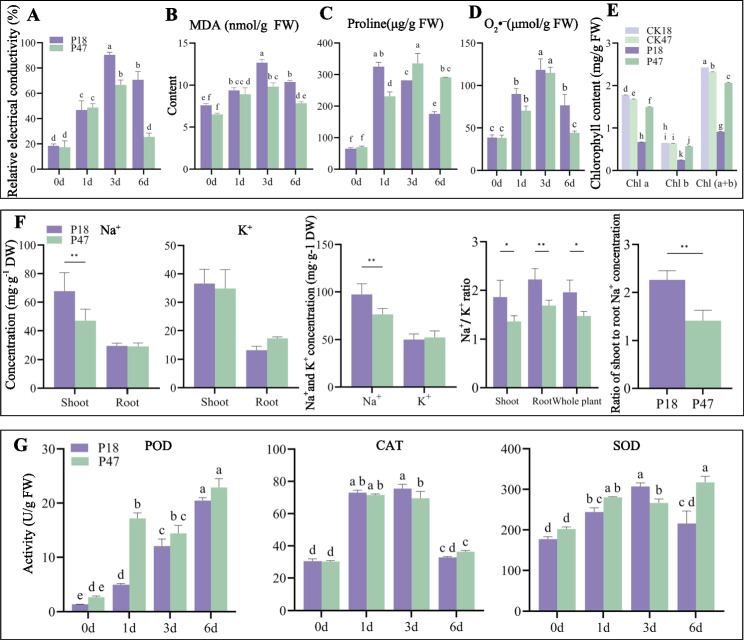
Physiological indicators of P18 and P47 under salt stress. **A** Relative conductivity. **B** Malondialdehyde content. **C** The variation in proline content. **D** The variation in O_2_• ^–^ content. **E** The variation in chlorophyll content. **F** The variation of Na^+^ and K^+^ in the aboveground parts and roots. **G** POD, CAT, SOD activity. Error bars indicate SD of three biological replicates. Different letters indicated significant difference between treatments. * *P* < 0.05, **P < 0.01; Significance level α = 0.05

Under salt stress, P18 exhibited 35.78 % higher relative electrical conductivity than P47 on day 3. By day 6, P18 showed significantly elevated conductivity versus day 0, while P47 reverted to baseline levels, indicating reduced membrane damage in P47 (Fig. 2A). MDA content increased by 36.59 % (P18) and 20.38 % (P47) after 6 days, with consistently higher accumulation in P18 (Fig. 2B) (Table S3).

Proline plays a significant role in maintaining osmotic balance in plants under salt stress [36]. After 1 day of treatment, the proline content in P18 increased rapidly but then continuously decreased with the extension of time. The increase in proline in P47 was relatively low, but it remained at a high level throughout (Fig. 2C) (Table S3). The content of O₂•⁻ in P18 was always higher than that in P47. At 6 days of stress, the content of O₂•⁻ in P18 was at a relatively high level, while that in P47 gradually approached the level at 0 days. The results indicated that the O₂•⁻ scavenging capacity of P47 was stronger than that of P18 (Fig. 2D) (Table S3).

After 6 days of salt stress, both P18 and P47 showed decreased chlorophyll content compared to controls. Specifically, P18 exhibited reductions of 62.41 % in chlorophyll a, 62.37 % in chlorophyll b, and 62.40 % in total chlorophyll. In contrast, P47 demonstrated decreases of 11.01 % in chlorophyll a, 11.11 % in chlorophyll b, and 11.04 % in total chlorophyll (Fig. 2E) (Table S3).

Ion homeostasis is critical for plant salt tolerance [37]. After 6-day salt stress, P47 maintained 30.56 % lower Na⁺ concentration in its aboveground parts than P18. The Na⁺/K⁺ ratios in P47 aboveground parts and roots were reduced by 24.20 % and 27.11 %, respectively, versus P18 (Fig. 2F). This indicates P47 restricts Na⁺ translocation to the aboveground parts through root ion sequestration, mitigating salt toxicity and enhancing tolerance. (Table S3).

Antioxidant enzymes help alleviate the ROS burst triggered by abiotic stress, thereby enhancing the plant's tolerance [38]. In this study, the activities of POD, CAT, and SOD were detected. The POD activity of P47 increased by 5.4 times after 1 day of salt stress, while that of P18 only increased by 2.5 times. After 6 days, the CAT activities of P18 and P47 increased by 6.94 % and 20.07 %, respectively. After 6 days, the SOD activity of P47 increased by 56.95 %, while that of P18 only increased by 21.79 %. This indicates that P47 has a stronger antioxidant capacity and can effectively alleviate oxidative damage (Fig. 2G) (Table S3).

### Observation of ultrastructure of P18 and P47 leaves under salt stress

**Fig. 3:**
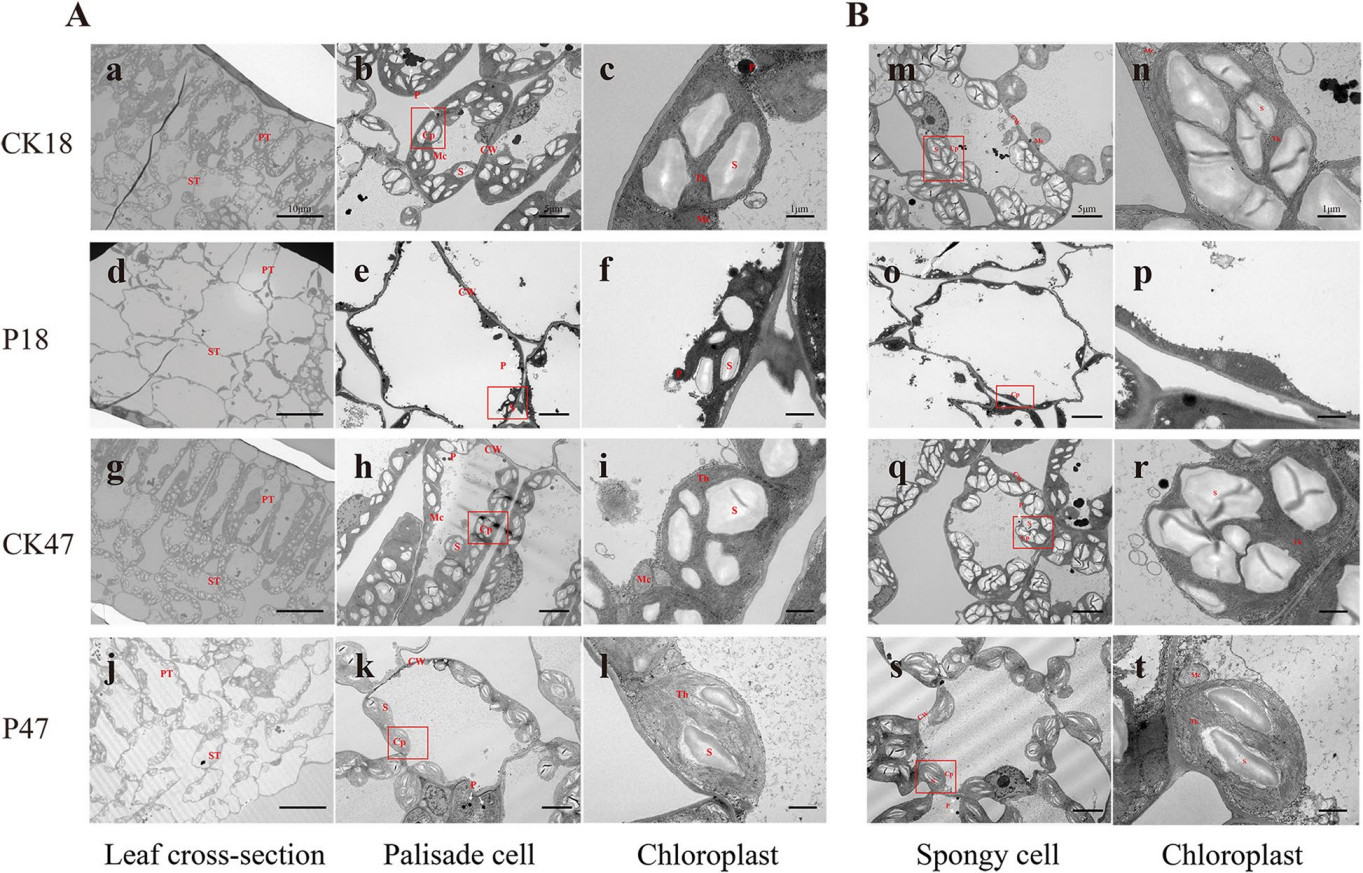
The effects of salt stress on the ultrastructure of chloroplasts in P18 and P47. **A** The overall arrangement of mesophyll, structure of palisade cell and chloroplast ultrastructure. The scales bar is 10 μm, 5 μm and 1 μm respectively. **B** Structure of spongy cell and chloroplast ultrastructure. The scales bar is 5 μm and 1 μm respectively. PT: Palisade tissue; ST: Spongy tissue; CW: Cell wall; Cp: Chloroplast; Mc: Mitochondria; S: Starch granules; P: Plastoglobulus; Th: Thylakoid

Salt stress severely disrupted P18 mesophyll cells after 6 days: palisade and spongy tissues became disorganized, chloroplast numbers decreased with membrane disintegration, and starch grains atrophied. In contrast, P47 maintained near-normal cellular architecture, with intact tissue arrangement and chloroplast ultrastructure (Fig. 3).

### Transcriptomic analysis of P18 and P47 in response to salt stress

**Fig. 4:**
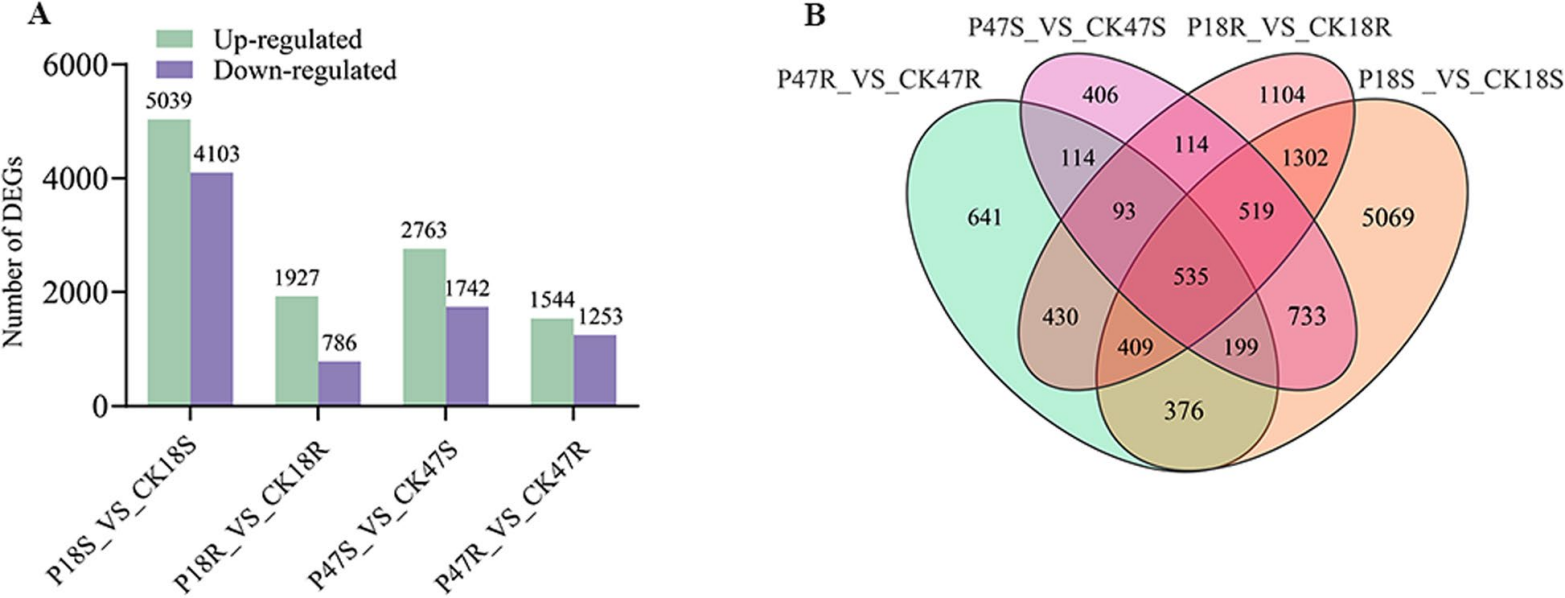
Analysis of DEGs. **A** Histogram of the number of DEGs. **B** Venn diagram of DEGs

Transcriptome sequencing was performed on shoot and root tissues of P18 and P47, along with their corresponding control groups, using the Illumina NovaSeq X Plus platform, generating 10.97 Gb of clean data, with Q30 bases ≥ 96.38 % (Table S4). Principal component analysis (PCA) demonstrated clear separation among treatment groups and tight clustering of biological replicates within groups, confirming the high reproducibility of the sequencing data (Fig S2). Comparisons were then made among different groups to screen for DEGs. Differential expression analysis identified 9,142 DEGs (5,039 upregulated; 4,103 downregulated) in P18S_VS_CK18S; 2,713 DEGs (1,927 up; 786 down) in P18R_VS_CK18R; 4,505 DEGs (2,763 up; 1,742 down) in P47S_VS_CK47S; and 2,797 DEGs (1,544 up; 1,253 down) in P47R_VS_CK47R (Fig. 4A). Venn analysis revealed 535 co-expressed DEGs across all comparison groups (Fig. 4B).

**Fig. 5:**
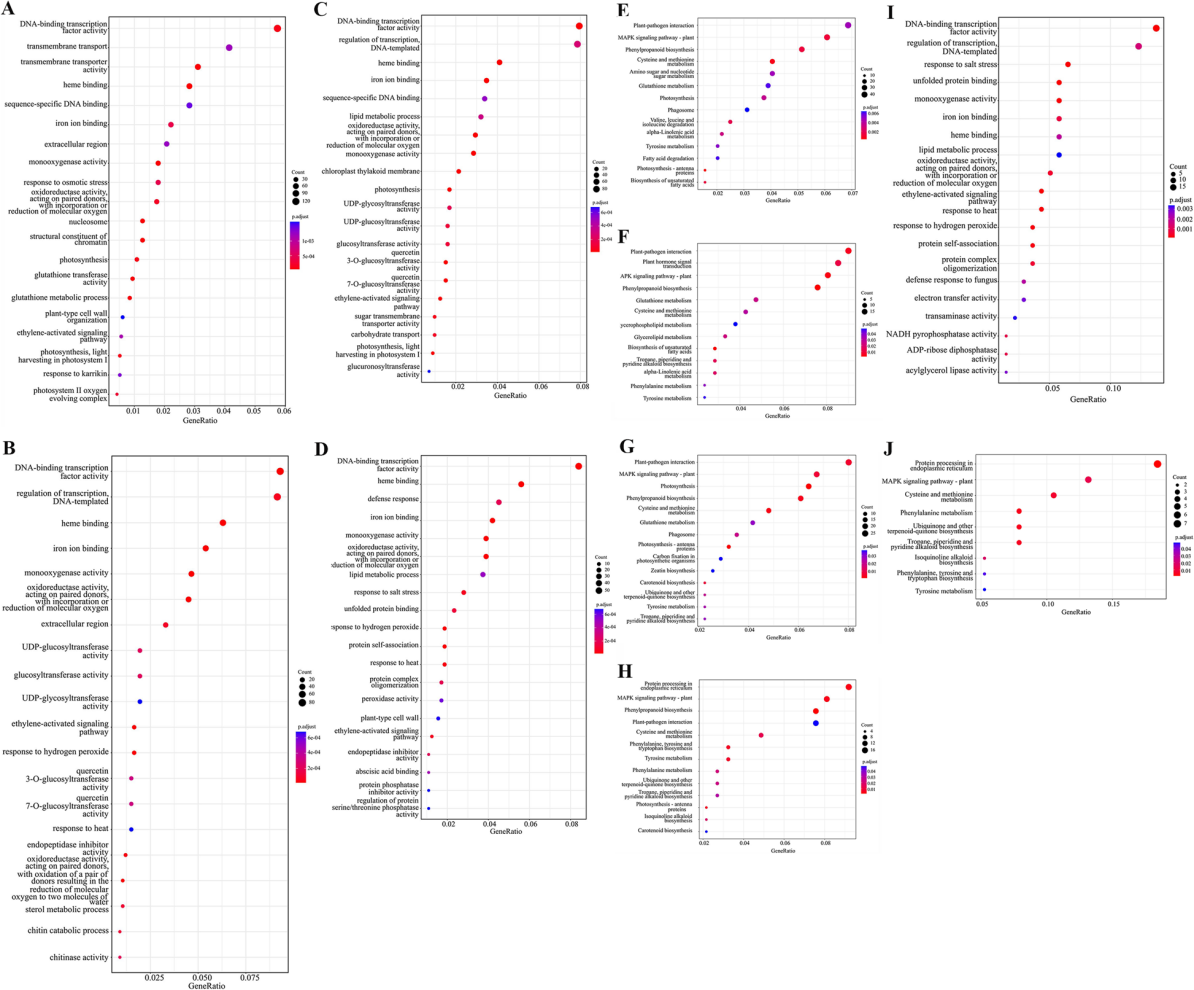
Bubble charts of GO and KEGG for DEGs. **A**-**D** GO enrichment bubble chart of DEGs. **E**-**H** KEGG enrichment bubble chart of DEGs. **I** GO enrichment bubble chart of DEGs in common. **J** KEGG enrichment bubble chart of DEGs in common

To further explore the functions of DEGs in pod pepper seedlings under salt stress, Gene Ontology (GO) and KEGG enrichment analyses were conducted. GO enrichment analysis revealed significant terms across the three standard categories: cellular component (CC), molecular function (MF), and biological process (BP) (Fig. S4). In terms of cellular components, P18 was mainly enriched in cell walls, nucleosome, photosystem II, while P47 was mainly enriched in extracellular region; in terms of molecular function, both were mainly enriched in DNA-binding transcription factor activity, glutathione transferase activity; in terms of biological process, they were mainly enriched in ethylene-activated signaling pathway, defense response, etc. (Fig. 5A-D). According to KEGG enrichment analysis, P18 was enriched in 24 pathways, mainly including MAPK signaling pathway-plant, Plant-pathogen interaction, Plant hormone signal transduction, and Carbon metabolism, P47 was enriched in 13 pathways, mainly including Phenylpropanoid biosynthesis, MAPK signaling pathway-plant, and Glutathione metabolism, etc. (Fig. 5E-H). GO enrichment analysis of the common DEGs revealed that in terms of molecular function, DEGs were mainly enriched in various enzyme activities such as oxidoreductase activity and phospholipase activity, and in biological processes, they were mainly enriched in lipid metabolism, amino acid metabolism, etc. (Fig. 5I). KEGG enrichment analysis of the common DEGs revealed that 9 pathways were enriched (Fig. 5J). The pathway with the highest number of enriched common DEGs was Protein processing in endoplasmic reticulum, followed by the MAPK signaling pathway - plant. Additionally, cysteine and methionine metabolism and phenylalanine metabolism pathways were significantly enriched. The enrichment of these pathways suggests their potential coordinated roles in regulating physiological responses, metabolic adjustments, and defense mechanisms in pepper seedlings under salt stress.

**Fig. 6:**
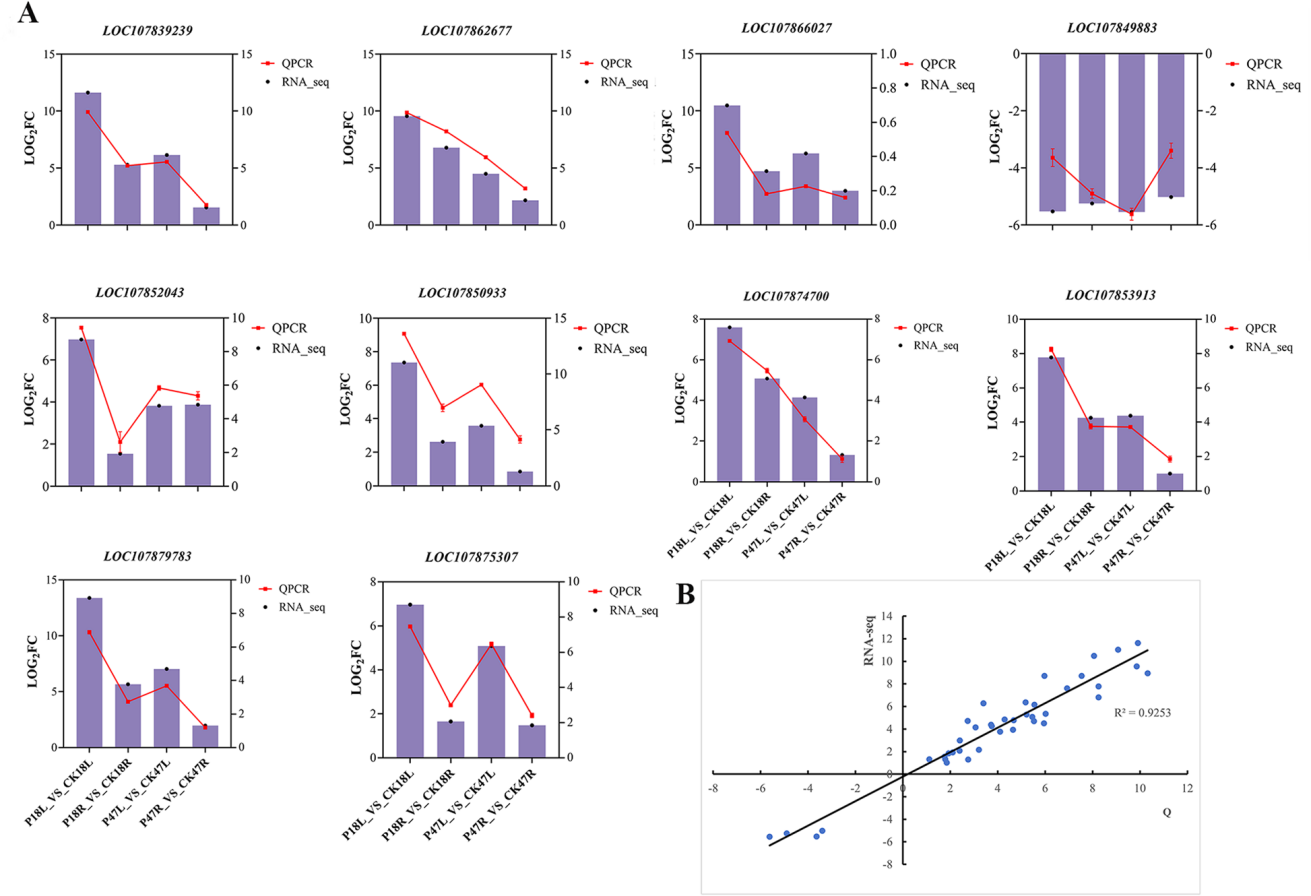
RT-qPCR verification. **A** RT-qPCR (broken line graph) and RNA-seq (histogram) results of genes in different comparison groups under salt stress. **B** Scatter plot was drawn using RT-qPCR data as X axis and RNA-seq data as Y axis, R^2^ = 0.9253

To validate the reliability of the RNA-seq results, the expression levels of ten randomly selected DEGs were assessed using RT-qPCR (Fig. 6). The RT-qPCR results demonstrated that the expression patterns of these selected DEGs were largely concordant with those observed in the RNA-seq data. The log2 fold changes measured by RT-qPCR and RNA-seq showed a strong positive correlation (R^2^ = 0.9253), supporting the reliability of the transcriptome data.

### Metabolomic analysis of P18 and P47 in response to salt stress

**Fig. 7:**
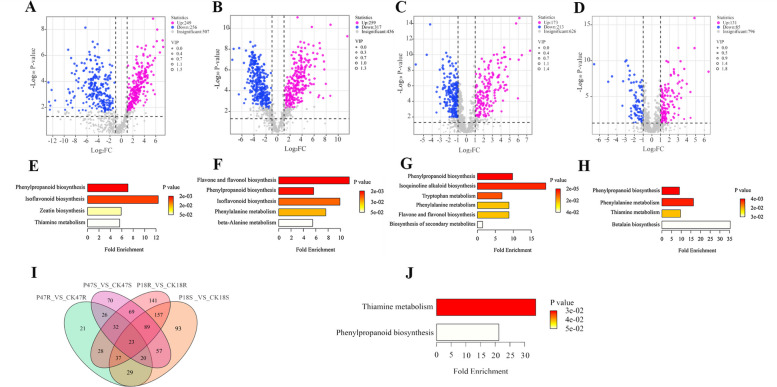
Metabolomic analysis of P18 and P47 in response to salt stress. **A**-**D** Metabolomic analysis of P18 and P47 in response to salt stress. **E** Venn diagram of differential metabolites. **F**-**I** Results of KEGG enrichment of different groups of differential metabolites. **J** Results of KEGG enrichment of common differential metabolites

To explore the metabolic basis of salt tolerance in pod pepper, non-targeted metabolomic analysis was conducted on the aboveground and root parts of P18, P47, and their respective control groups. Orthogonal partial least squares discriminant analysis (OPLS-DA) was used to construct a relationship model between the metabolite content and sample categories of each group, revealing significant differences in metabolites before and after salt treatment (Fig. S3). DAMs analysis identified 505, 576, 386, and 216 DAMs, respectively (Fig. 7A-D). Venn analysis detected 23 common DAMs across all groups (Fig. 7I), including terpenoids, homoterpenes, and vinca alkaloids. KEGG enrichment analysis was performed on the identified DAMs (Fig. 7E-H). In P18S_VS_CK18S, phenylpropanoid biosynthesis, isoflavonoid biosynthesis, zeatin biosynthesis, and thiamine metabolism pathways were significantly enriched. In P18R_VS_CK18R, flavonoid biosynthesis, phenylpropanoid biosynthesis, isoflavonoid biosynthesis, phenylalanine metabolism, and β-alanine metabolism pathways were enriched. In P47S_VS_CK47S, phenylpropanoid biosynthesis, isoquinoline alkaloid biosynthesis, tryptophan metabolism, phenylalanine metabolism, flavonoid and flavonol biosynthesis, and biosynthesis of secondary metabolites pathways were enriched. In P47R_VS_CK47R, phenylpropanoid biosynthesis, phenylalanine metabolism, thiamine metabolism, and Glycine, serine and threonine metabolism pathways were significantly enriched. KEGG enrichment analysis of the common DAMs among the four groups only identified two metabolic pathways: thiamine metabolism and phenylpropanoid biosynthesis (Fig. 7J).

### Correlation analysis between key metabolites and DEGs

**Fig. 8:**
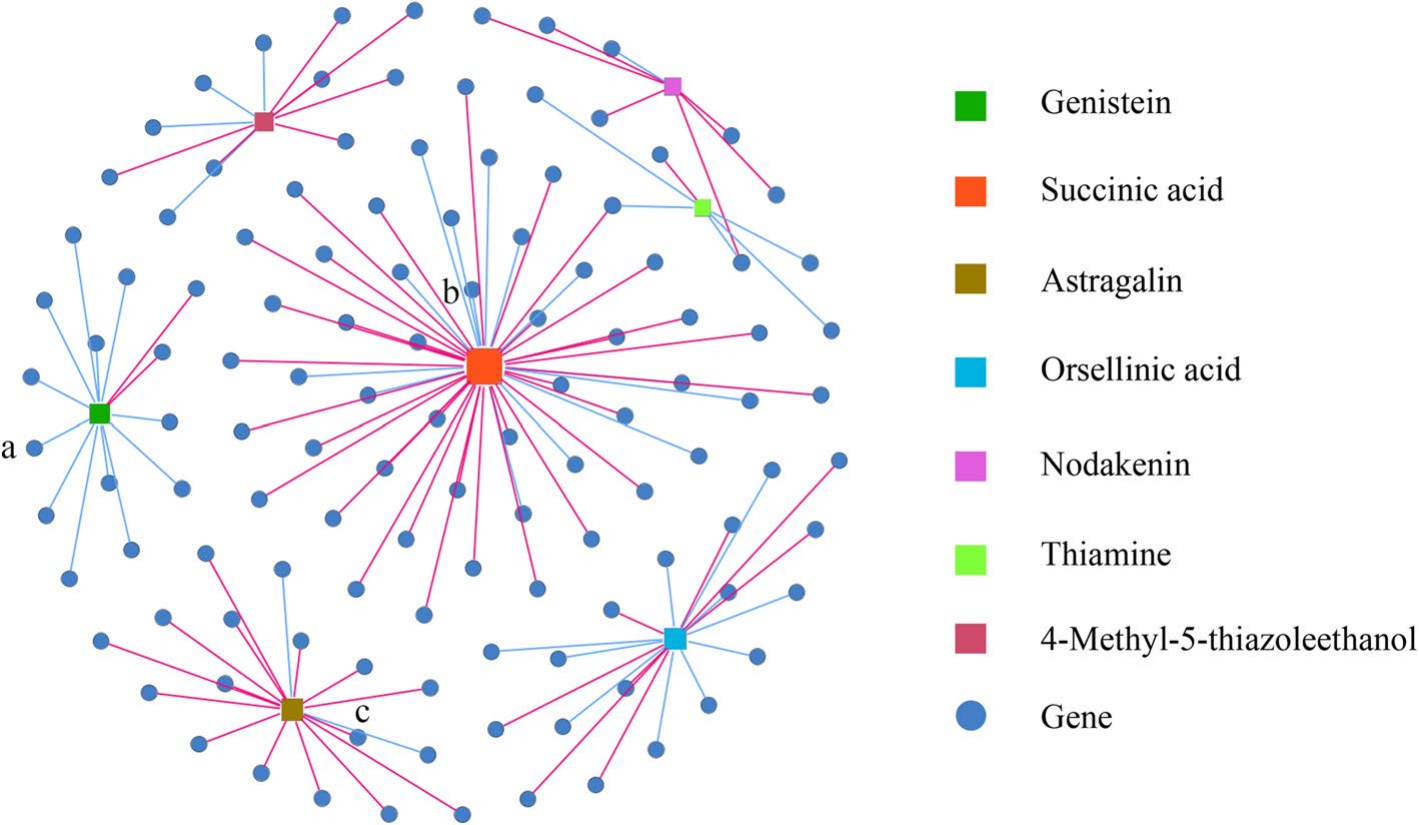
Metabolite-gene co-regulation network. **a** *LOC107867623*, **b** *LOC107873120*, **c** *LOC107840165*. Colored squares denote the seven key metabolites, while circles indicate correlated genes. Blue edges indicate positive correlations, and red edges indicate negative correlations. (Pearson's |R| >0.08, adjusted *P* < 0.01; edge weight >0.8)

The DAMs exhibiting the most significant alterations in all groups were selected as key metabolites, resulting in the identification of seven key metabolites (genistein, succinic acid, astragalin, orsellinic acid, nodakenin, thiamine, 4-methyl-5-thiazoleethanol). Correlation analyses between the seven key metabolites and DEGs revealed that 121 DEGs were strongly correlated with the key metabolites (|R| >0.08, *P* < 0.01) (Table S5). Notably, node centrality analysis highlighted succinic acid as the most influential metabolite, demonstrating the highest connectivity within the co-regulation network, followed by orsellinic acid and astragalin. Specifically, genistein was positively correlated with 12 genes and negatively correlated with 2 genes, including *LOC107867623* in the SDR family; succinic acid was positively correlated with 14 genes and negatively correlated with 34 genes, including *LOC107873120* in the ADH family; astragalin was positively correlated with 2 genes and negatively correlated with 15 genes; orsellinic acid was positively correlated with 10 genes and negatively correlated with 8 genes; nodakenin was positively correlated with 1 gene and negatively correlated with 6 genes; thiamine was positively correlated with 5 genes and negatively correlated with 1 gene, including *LOC107840165* in the pathogenesis-related (PR) protein gene family; 4-methyl-5-thiazoleethanol was positively correlated with 4 genes and negatively correlated with 7 genes (Fig. 8).

### DEGs involved in different metabolic pathways

**Fig. 9:**
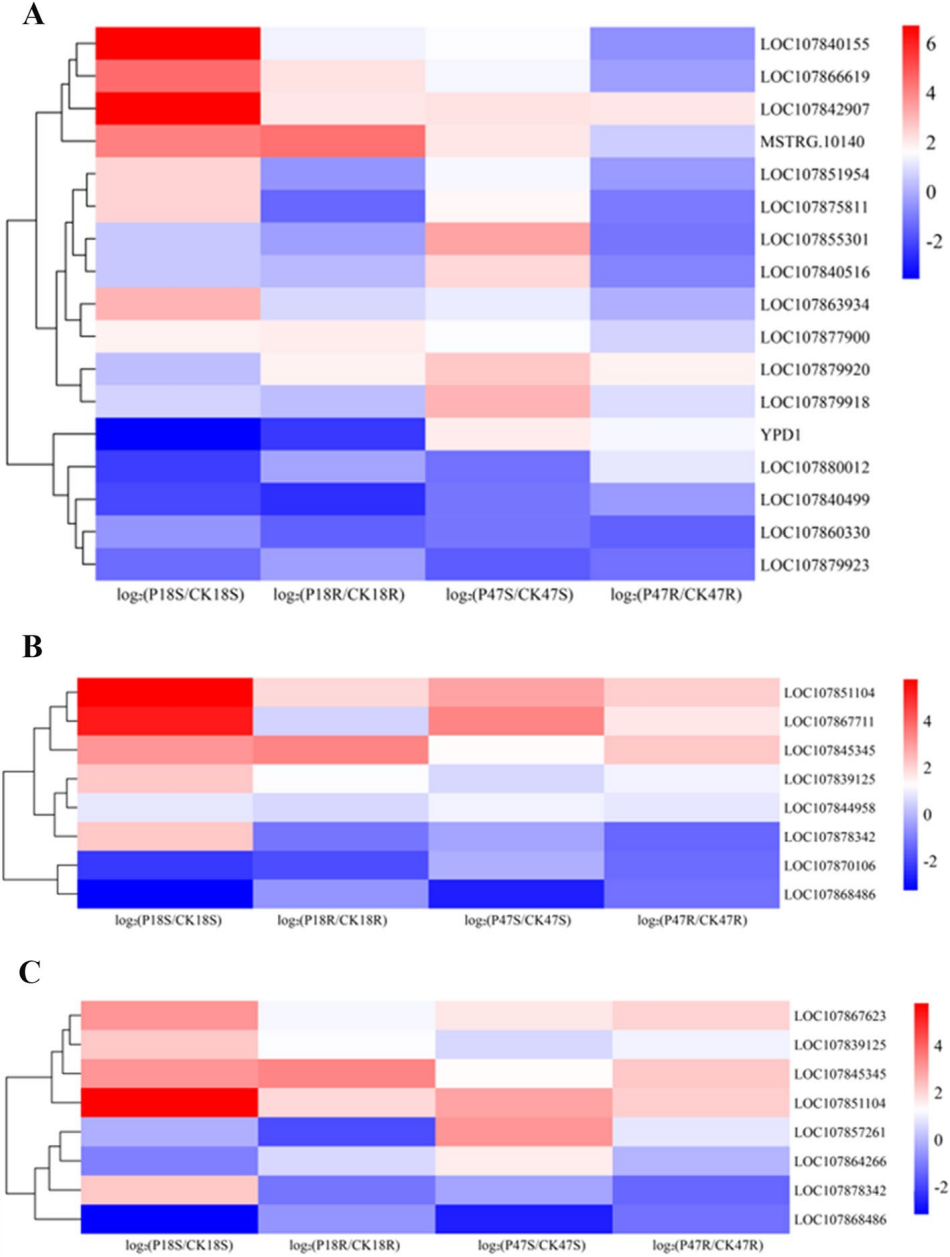
**A** Heat map analysis of genes related to hormone signal transduction. **B** Heat map analysis of genes related to tyrosine metabolism. **C** Heat map analysis of genes related to alkaloid synthesis

A total of 18 DEGs associated with plant hormone signal transduction pathways were identified in P47, comprising 14 up-regulated and 4 down-regulated genes (Fig. 9A). These DEGs are primarily involved in the auxin (IAA), jasmonic acid (JA), abscisic acid (ABA) and ethylene (ETH) signaling pathways, regulating the balance between plant growth and stress defense. Among these, four up-regulated DEGs belong to the PR protein gene family. Notably, *LOC107879920* and *LOC107879918* exhibited high fold changes in P47 leaves, with increases of 2.698-fold and 3.0997-fold, respectively. Auxin-responsive genes, including members of the SAUR and ARF families (e.g., *ARF7*), were also significantly up-regulated, suggesting their potential key roles in mediating salt stress responses in P47.

Tyrosine metabolism plays a significant role in plant salt stress responses, contributing to the generation of phenolic compounds, osmolytes, and plant hormones [39]. A total of 8 DEGs associated with tyrosine metabolism were identified, including *ADH1. ADH1* expression was upregulated across all analyzed conditions, with fold changes of 5.244 and 3.416 in P18S vs CK18S and P47S vs CK47S, respectively (Fig. 9B). The up-regulation of these genes suggests their potential contribution to the plant's salt stress response and tolerance.

Alkaloids contribute to plant tolerance to salt stress [40]. A total of 8 DEGs associated with the alkaloid biosynthetic pathway were identified, among which 5 were also associated with tyrosine metabolism. This overlap indicates that these genes function in multiple metabolic pathways during the salt stress response. *LOC107857261* encodes *putrescine N-methyltransferase 1* (*PMT1*) and was upregulated specifically in P47 leaves, exhibiting a 3.121-fold increase. *LOC107867623* encodes tropinone reductase and was significantly upregulated in both roots (2.0-fold) and leaves (1.5-fold), suggesting that it may contribute to mitigating salt stress damage by promoting alkaloid accumulation (Fig. 9C).

## Discussion

Pepper seedlings exhibit high sensitivity to salt stress, severely restricting large-scale cultivation and yield improvement in saline-alkaline soils [41]. Salt-tolerant germplasms serve as valuable genetic resources for enhancing crop salt tolerance. Consequently, deciphering salt tolerance mechanisms remains a critical research priority. Herein, a salt-tolerant genotype (P47) and a salt-sensitive genotype (P18) were identified through multi-level phenotypic screening. By characterizing their differential responses to salt stress across physiological, biochemical, transcriptional, and metabolic levels, this integrated approach elucidates salt stress impacts on plant physiology and reveals the molecular basis of genotypic divergence in salt tolerance.

The accumulation of osmotic adjustment substances in plants contributes to the maintenance of ion and osmotic homeostasis, thereby mitigating salt stress-induced cellular damage [42]. Proline functions not only as an osmoticum but also as a ROS scavenger and protein stabilizer. Variation in its content partially explains the differential salt tolerance observed among plant varieties [43]. Maintaining a high K⁺/Na⁺ ratio and low Na⁺ accumulation in shoots are key determinants of plant salt tolerance [44]. In this study, P47 sustained a high proline concentration, which helped stabilize cellular osmotic potential and enzyme activity. In contrast, P18 exhibited impaired proline synthesis during prolonged stress, likely due to cellular damage. Notably, P47 exhibited lower Na⁺ concentrations in both roots and shoots compared to P18. Furthermore, P47 maintained relatively high Na⁺ retention in its roots, suggesting mechanisms such as restricted xylem loading and root Na⁺ sequestration to limit Na⁺ translocation to the shoots, thereby alleviating shoot damage. Salt stress triggers excessive ROS production, causing oxidative damage and physiological dysfunction [45]. Antioxidant enzyme activities (POD, CAT, SOD) declined in P18 under prolonged salt treatment, compromising its ROS scavenging capacity and resulting in a vicious cycle of oxidative damage. Conversely, P47 maintained higher POD, CAT, and SOD, enabling continuous ROS detoxification and preventing the accumulation of oxidative injury.

Plant hormones play critical regulatory roles in plant growth, development, and responses to various stresses. Multiple phytohormones coordinate to regulate physiological and biochemical processes in plants. In this study, P47 and P18 exhibit sharply divergent regulatory networks. P47 activated multiple hormones signaling pathways IAA, JA, ABA, and ETH to coordinately regulate the stress response. This coordinated activation was accompanied by upregulated expression of auxin-related genes *SAUR* and *ARF7.* Among these, *SAUR* (*Small Auxin Up RNA*) belongs to the early auxin response genes, which can be rapidly activated and transcribed after auxin induction without the need for new protein synthesis [46]. This gene family can promote cell elongation by regulating cell wall acidification and alleviating growth inhibition caused by salt stress. For example, overexpression of *AbSAUR1* in *Atropa belladonna* showed multiple phenotypic changes such as increased plant height, branch number, and biomass, exerting a positive regulatory effect on plant growth and development [47]. ARF7 (Auxin Response Factor 7), as a core transcription factor in auxin signaling, can activate downstream target genes, promote auxin polar *transport*, and play a key role in lateral root development [48]. Thus, P47 may alleviate stress-induced growth inhibition by upregulating *SAUR* and *ARF7* to promote cell elongation and lateral root development.

The synthesis of alkaloids is closely related to tyrosine metabolism, and many genes can simultaneously participate in both metabolic pathways and play different roles. For instance, dopamine generated from the tyrosine metabolic pathway serves as a precursor for certain alkaloids [49]. Many genes achieve the transition from short-term stress to long-term adaptation by balancing alkaloid and tyrosine metabolism. PMT is the first key rate-limiting enzyme in the synthesis pathway of tropane and nortropane alkaloids, catalyzing the conversion of putrescine to N-methylputrescine. N-methylputrescine serves as the starting point for the formation of the tropane alkaloid skeleton, which is subsequently acted upon by enzymes like tropinone reductase (TR) to generate specific tropane alkaloids [50]. In this study, the sustained upregulation of the *TAT2* gene in P47 facilitated the conversion of tyrosine to dopamine, providing precursors for tropane alkaloid biosynthesis. Concurrently, the coordinated expression of rate-limiting enzyme genes such as *PMT* and *TR* established the complete biosynthetic pathway from putrescine to specific tropane alkaloids. This regulatory mechanism was not significantly activated in P18, suggesting that alkaloid metabolic reprogramming may represent a unique salt tolerance strategy specific to P47.

Furthermore, P47 demonstrated enhanced metabolic plasticity by activating multiple pathways including flavonoids (e.g., genistein), betaine, and tryptophan metabolism, establishing a multi-dimensional defense network. The sustained upregulation of key genes such as *TAT* and *alcohol dehydrogenase* (*ADH*) further strengthened its regulatory advantage in secondary metabolism and redox balance.

In summary, the salt-tolerant genotype P47 establishes a multi-layered responsive network by integrating ion compartmentalization, antioxidant defense, hormone signal transduction, and secondary metabolic reprogramming. In contrast, P18 exhibits sensitivity due to insufficient regulation of these key pathways. This study provides the first systematic demonstration of the significant role of the alkaloid biosynthesis pathway in pepper's response to salt stress, offering new targets and a theoretical foundation for molecular design breeding of salt-tolerant pepper varieties.

## Conclusion

This study comprehensively analyzed the physiological indices, transcriptome, and metabolome of salt-tolerant variety P47 and salt-sensitive variety P18 pod pepper seedlings under salt stress. The results showed that P47 exhibited significant salt tolerance advantages under salt stress. The Na^+^/K^+^ratio, electrical conductivity, malondialdehyde, and O₂•⁻ content in P47 were significantly lower than those in P18. Concurrently, P47 exhibited higher activities of antioxidant enzymes and accumulated more proline, effectively alleviating oxidative damage and enhancing osmotic regulation. Multi-omics data revealed enrichment of phenylpropanoid, isoflavonoid, and alkaloid biosynthesis pathways, with significant alterations in key metabolites (e.g., kaempferol, quercetin) and genes (e.g., *SAUR*, *ARF7*, *TAT*, *PMT*), suggesting their synergistic role in salt tolerance. The two varieties employed distinct response mechanisms: P47 demonstrated significant enrichment in the glutathione metabolism and MAPK signaling pathway-plant, whereas P18 exhibited greater disruption to its photosynthetic and energy metabolism processes. Although both varieties activated shared stress-responsive pathways, distinct varietal differences were evident in their specific regulatory mechanisms and physiological outcomes. These findings suggest that the enhanced salt tolerance of P47 may be attributed to its robust antioxidant capacity and efficient signal transduction, whereas the sensitivity of P18 likely stems from impairments in energy metabolism. These results reveal the differential regulatory mechanisms underlying salt tolerance in pod peppers and lay a theoretical foundation for breeding resistant cultivars through genetic improvement.

## Supplementary Information

Supplementary Material 1: Table S1. The numbers and names of the tested materials. Table S2. Salt damage index of different cultivar of pod pepper. Table S3. Physiological indicators of P18 and P47 under salt stress. Table S4. Transcriptome sample sequencing data statistics. Table S5. Primers used for RT-qPCR. Table S6. DEGs strongly associated with key metabolites.

Supplementary Material 2: Fig. S1. Temporal phenotypic responses of pod pepper seedlings to salt stress. Fig. S2. PCA score plots of metabolic profiles. Fig. S3. Sample OPLS-DA score plot and permutation test. Fig. S4. GO enrichment classification of differentially expressed genes.

## Data Availability

The data have been publicly available. The metabolomic data are accessible from the NGDC OMIX database under accession number OMIX012518 (https://ngdc.cncb.ac.cn/omix/release/OMIX012518), and the RNA-seq data are available from the NGDC GSA under accession number CRA032258 (https://ngdc.cncb.ac.cn/gsa/browse/CRA032258).

## Abbreviations

ROS: Reactive oxygen species
SOD: Superoxide dismutase
POD: Peroxidase
PHt: Plant height
FW: Fresh weight
DW: Dry weight
RPHt: Relative plant height
RFW: Relative fresh weight
RDW: Relative dry weight
D value: Comprehensive salt-tolerance evaluation value
STI: Salt tolerance index
ICP-MS: Inductively coupled plasma mass spectrometry
TEM: Transmission electron microscopy
Pro: Proline
O₂•⁻: Superoxide anion
H₂O₂: Hydrogen peroxide
MDA: Malondialdehyde
TBA: Thiobarbituric acid
CAT: Catalase
DEGs: Differentially expressed genes
RT-qPCR: Reverse transcription quantitative real-time PCR
DAMs: Differentially accumulated metabolites
OPLS-DA: Orthogonal partial least squares discriminant analysis
Nr: NCBI non-redundant protein sequences
Nt: NCBI non-redundant nucleotide sequences
Pfam: Protein family
KOG/COG: Pods of Orthologous Groups of proteins
Swiss-Prot: A manually annotated and reviewed protein sequence database
KO: KEGG Ortholog database
GO: Gene ontology
CC: Cellular component
MF: Molecular function
BP: Biological process
PR: Pathogenesis-related
IAA: Auxin
JA: Jasmonic acid
ETH: Ethylene
TAT: Tyrosine aminotransferase
ADH: Alcohol dehydrogenase
TR: Tropinone reductase
