# Supplementary material for: Physiological and biochemical variations in different pepper (Capsicum annuum var. conoides) varieties under salt stress
Source: BMC Plant Biol. 2025 Dec 8;26:67. doi: 10.1186/s12870-025-07873-0 (PMC12798108; doi:10.1186/s12870-025-07873-0)
Supplement: Supplementary file 2 — Supplementary Material 2: Fig. S1. Temporal phenotypic responses of pod pepper seedlings to salt stress. Fig. S2. PCA score plots of metabolic profiles. Fig. S3. Sample OPLS-DA score plot and permutation test. Fig. S4. GO enrichment classification of differentially expressed genes. [file 12870_2025_7873_MOESM2_ESM.pdf]

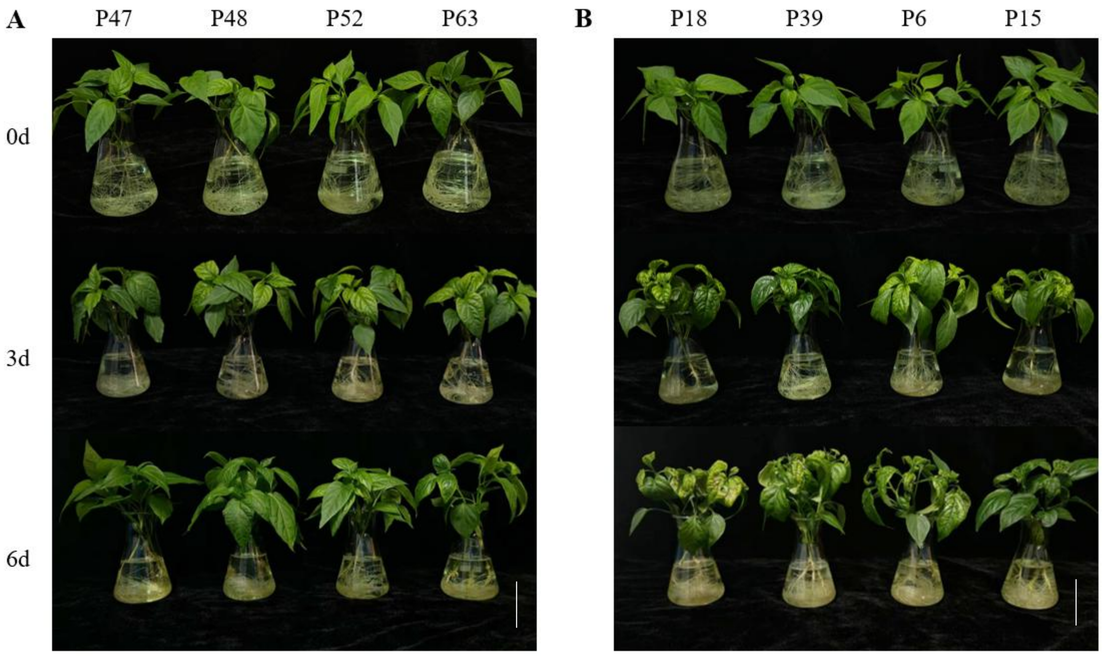

Fig. S1 Temporal phenotypic responses of pod pepper seedlings to salt stress. (A) Four cultivars of salt-tolerant pod pepper. Scale bar = 8 cm. (B) Four cultivars of salt-sensitive pod pepper. Scale bar = 8 cm.

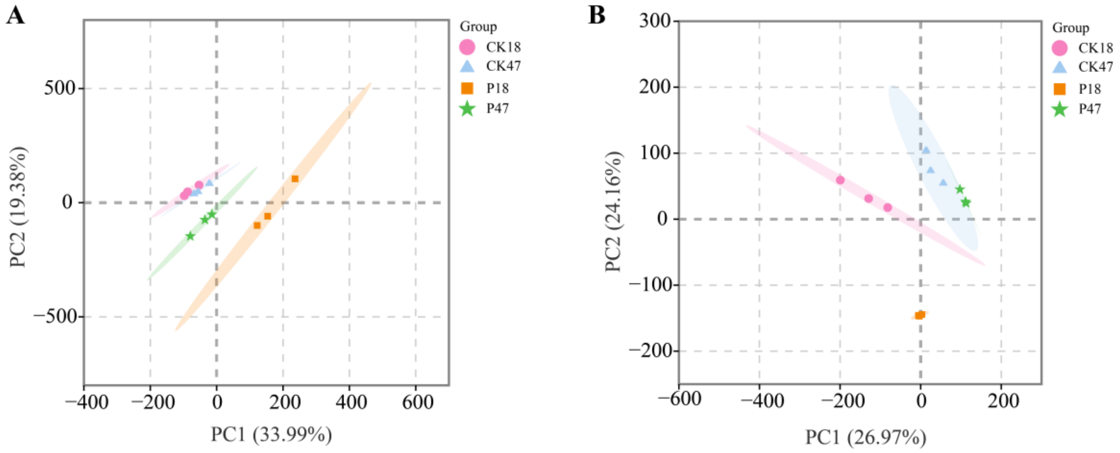

Fig. S2 PCA score plots of metabolic profiles. (A) PCA diagram of the shoot of each component. (B) PCA diagram of the root of each component.

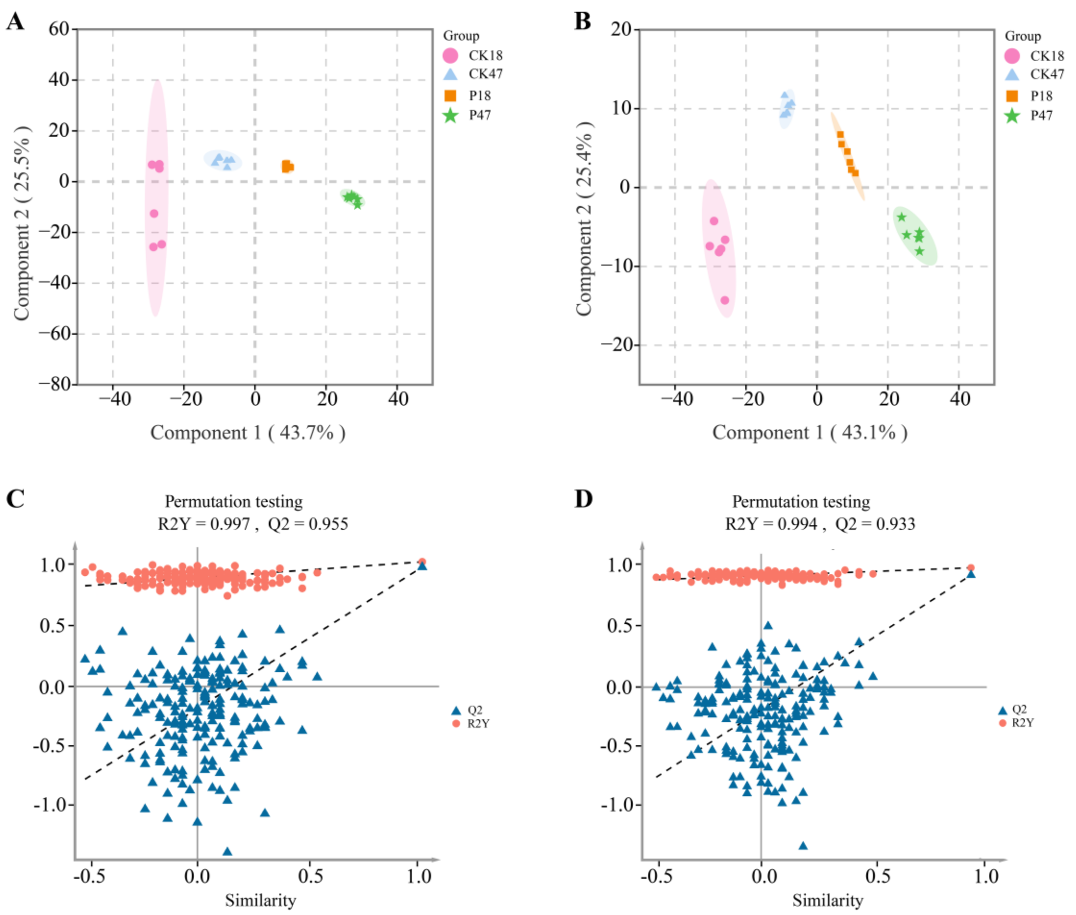

Fig. S3 Sample OPLS-DA score plot and permutation test. (A) OPLS-DA score plot of the shoot grouping. (B) PCA diagram of the root of grouping. (C) Evaluation of displacement test for the shoot grouping. (D) Evaluation of displacement test for the root grouping.

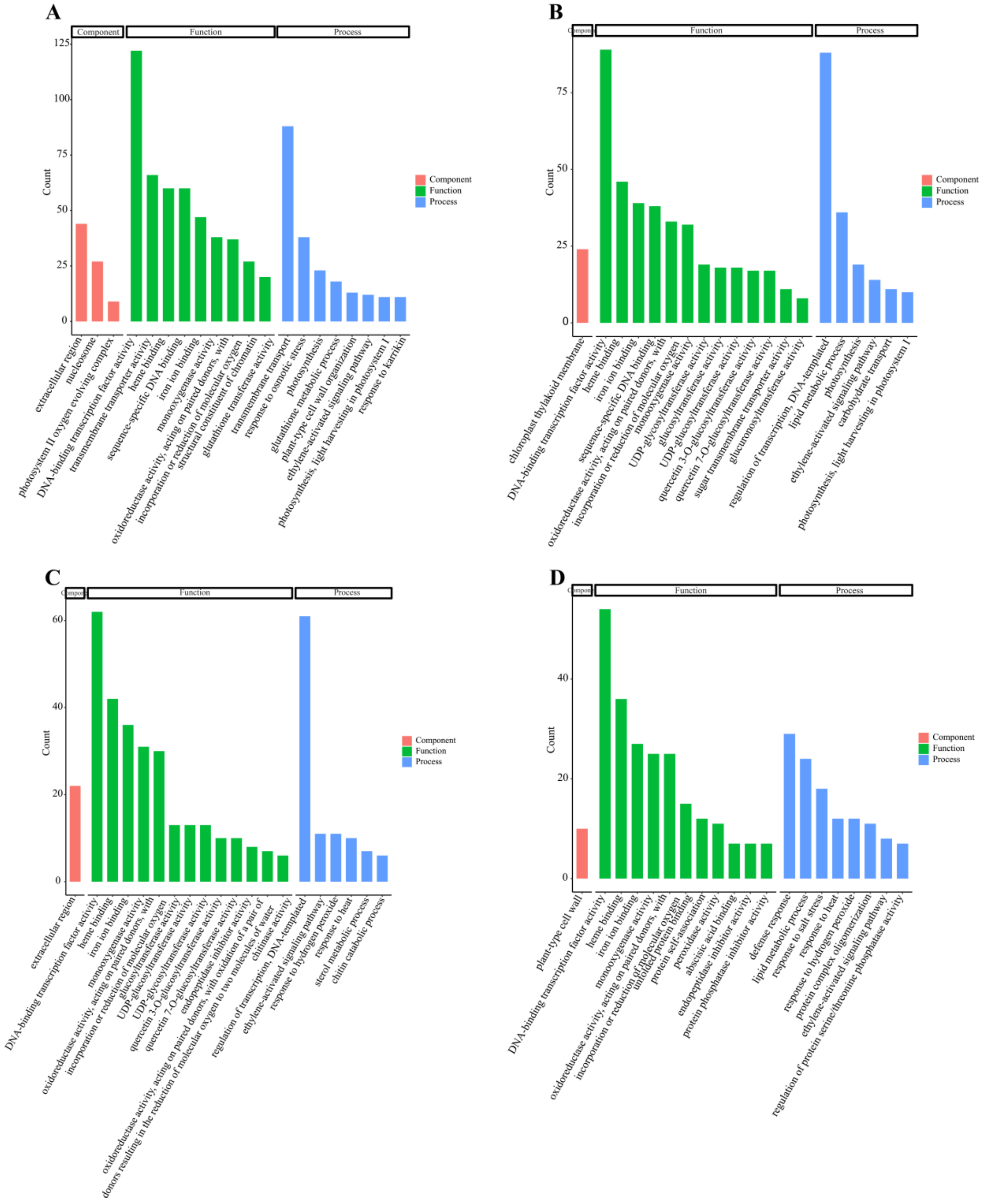

Fig. S4 GO enrichment classification of differentially expressed genes. (A-B) GO enrichment of P18_VS_CK18 differentially expressed genes in the shoot and root. (C-D) GO enrichment classification map of P47_VS_CK47 differentially expressed genes in shoot and root.
